# Thyroid dysfunction and cognitive function: a review

**DOI:** 10.3389/fendo.2026.1843431

**Published:** 2026-08-03

**Authors:** HongShi Shi, YingYing Han, YueNan Zheng, MengYuan Zhang, MoriGen Wang, MoFei Wang

**Affiliations:** 1 Department of Thyroid, Breast and Hernia Surgery, Affiliated Hospital of Inner Mongolia Minzu University, Tongliao, China; 2 College of Clinical Medicine, Inner Mongolia Minzu University, Tongliao, China

**Keywords:** cognitive dysfunction, cognitive impairment, executive function, memory, thyroid function, thyroid hormone

## Abstract

Recently, the incidence of thyroid diseases has been on the rise. While standardized diagnosis and treatment generally yield excellent prognoses, the association between thyroid disorders and cognitive function remains a topic of significant interest. Cognitive function includes memory, language, visuospatial ability and executive function. Cognitive dysfunction refers to the impairment of one or more of the above, including mild cognitive impairment (MCI) and dementia. More and more studies have shown that thyroid disease is associated with cognitive dysfunction. Clinically, some patients present with noticeable symptoms such as memory loss and inattention, which severely impede their daily social functioning. There is a consensus on the view that thyroid hormone deficiency during the neonatal or embryonic period can cause disorders in the central nervous system, resulting in severe cognitive impairment such as persons with congenital hypothyroidism, but there is disagreement on the relationship between adult thyroid diseases, including clinical hyperthyroidism, hypothyroidism, subclinical hyperthyroidism, subclinical hypothyroidism and cognitive dysfunction. The inconsistencies in research conclusions are attributed to complex factors. Differences in age stratification constitute an important confounding factor for thyroid-related cognitive impairment; various neuropsychological assessment tools show obvious discrepancies in the sensitivity of detecting cognitive damage, which can directly affect the evaluation of research outcomes. Meanwhile, methodological heterogeneity among studies is also a key inducement for the inconsistent findings on the association between clinical thyroid dysfunction and cognitive function in adults. Therefore, this article mainly reviews the relationship and research progress between thyroid function and cognitive dysfunction in adults. From this review, we conclude that obvious thyroid dysfunction is obviously related to cognitive function, mainly focusing on attention and memory. Treatment would improve cognition to some extent, but wouldn’t fully restore it. The relationship between subclinical thyroid dysfunction and cognitive function is still of important significantly.

## 1 Introduction

Cognitive impairment is defined as poor performance in one or more cognitive domains, including memory, attention, executive function, and visual-spatial ability, and dementia represents a more severe form of cognitive impairment (1). There are about 50 million dementia patients worldwide, and about 10 million new cases every year (2). The high incidence of dementia and cognitive impairment exerts a huge impact on individuals, families, and the social economy, thereby significantly increasing the social burden (3). The incidence of endocrine system diseases is increasing year by year. Since the nervous system and the endocrine system are closely interrelated, research has shown that endocrine disorders have a certain impact on the central nervous system (4). Thyroid dysfunction is a common endocrine system disease, it is been confirmed as one of the risk factors for cognitive impairment by several mechanism in several aspects (5). It is involved in various processes, including the occurrence, development, and maturation of the nervous system, thus the changes in thyroid hormones can affect synaptic plasticity and neurogenesis, further exerting a significant impact on cognitive abilities, including learning and memory (6). Thyroid hormones (THs) mainly consist of thyroxine (T4) and triiodothyronine (T3). T4 serves as a circulating prohormone and is converted into biologically active T3 by iodothyronine deiodinases. Thyroid hormone receptors (TRs) are widely distributed in brain tissues and mediate the physiological actions of THs during brain development. Notably, the complete TH signaling machinery, including deiodinases and TRs, has been detected in the fetal brain long before the fetal thyroid gland becomes functional. This evidence strongly indicates that maternal thyroid hormone plays an irreplaceable and critical role in early fetal brain development (7, 8). In recent years, the incidence of thyroid disease is rising, the prognosis is excellent with standardized diagnosis and treatment. Cognitive function includes memory, language, visuospatial ability and executive function. Cognitive dysfunction refers to the impairment of one or more of the above, including mild cognitive impairment (MCI) and dementia. A growing body of evidence has shown that thyroid disease is associated with cognitive dysfunction. It has been observed that some patients may experience memory loss and inattention, which seriously affect daily social functioning (9). Therefore, this association has received widespread attention. There is a consensus that thyroid hormone deficiency during the neonatal or embryonic period can cause disorders in the central nervous system, resulting in severe cognitive impairment such as persons with congenital hypothyroidism (4, 6). However, there is disagreement on the relationship between adult thyroid diseases, including clinical hyperthyroidism, hypothyroidism, subclinical hyperthyroidism, subclinical hypothyroidism and cognitive dysfunction. Therefore, this article mainly reviews the relationship and research progress between thyroid function and cognitive dysfunction in adults. From this review, we conclude that overt thyroid dysfunction is significantly associate to cognitive function, primarily affecting attention and memory. Treatment can improve cognition to some extent, but cannot fully restore it. The relationship between subclinical thyroid dysfunction and cognitive function is still of important significantly. Thus, in this review, we summarized the relationships between thyroid dysfunction and subclinical thyroid dysfunction to enhance understanding of this topic.

## 2 Methods

This narrative review aims to examine the relationship and research progress between thyroid dysfunction and cognitive impairment in adults. To ensure a comprehensive, objective, and scientifically rigorous assessment, we systematically searched electronic databases, including PubMed/MEDLINE, Web of Science, Cochrane Library, and Embase from their inception through December 2025. The search strategy combined subject headings and free-text terms, incorporating both Chinese and English keywords such as thyroid dysfunction, hypothyroidism, subclinical hypothyroidism, hyperthyroidism, subclinical hyperthyroidism, thyroid hormones, cognitive function, cognitive impairment, mild cognitive impairment, dementia, memory, executive function, and hippocampus. Two authors independently performed the initial screening of titles and abstracts, followed by a full-text review. Any discrepancies in screening decisions were resolved through discussion between the two reviewers, with the final determination made by the corresponding author. After this tiered screening process, 70 high1-quality studies were included, encompassing clinical cohorts, controlled intervention trials, animal studies, meta−analyses, and relevant reviews. Extracted data comprised basic study information, participant characteristics, cognitive assessment tools and their measured domains, main findings with statistical effect sizes, as well as treatment interventions and their effects on cognitive function. Given the substantial heterogeneity among the included studies, including differences in study populations, cognitive assessment tools, definitions of thyroid function, and TSH cut-off values, a quantitative meta-analysis was not conducted. Instead, a narrative synthesis approach was employed to summarize the effects of thyroid dysfunction on cognitive function, stratified by type of thyroid dysfunction. The synthesis focused on characterizing impairments across different cognitive domains, examining cognitive changes before and after treatment, and exploring potential reasons for inconsistencies in the findings across studies.

## 3 Molecular mechanisms underlying TH regulation of cognitive function

Thyroid hormones (THs) modulate memory, synaptic plasticity and multiple cognitive domains via genomic and non-genomic signaling pathways. The hippocampal CA1 region, enriched with thyroid hormone receptors (TRs), is the core target of TH action in the brain. At the molecular level, biologically active T3 binds to nuclear TRα and TRβ to regulate the transcription of downstream genes (10). TH deficiency or excess downregulates the expression of brain-derived neurotrophic factor (BDNF) and impairs the BDNF/TrkB cascade, which is essential for neuronal survival and neurogenesis (11). In terms of synaptic plasticity, TH disorders alter the expression and function of AMPA and NMDA glutamate receptors on postsynaptic membranes, further affecting long-term potentiation (LTP), the cellular basis of learning and memory (12). The cAMP-CREB-MAPK/ERK signaling pathway, closely linked to memory consolidation, is also markedly suppressed under abnormal TH status (13). Additionally, TH fluctuations affect acetylcholine synthesis, Na^+^,K^+^-ATPase activity and glutamatergic transmission (14). Abnormal TH levels also trigger excessive Tau phosphorylation and amyloid precursor protein accumulation, accelerating neuronal damage (15). Notably, overt and subclinical thyroid dysfunction differ in molecular manifestations. Overt TH disturbance causes severe damage to dendritic spines and irreversible signaling deficits, while subclinical thyroid disorders mainly induce mild alterations in neurotransmitter balance and neural circuit connectivity, predominantly impairing executive function and information processing speed (9).

### 3.1 Overt hypothyroidism and cognition

**Figure 1:**
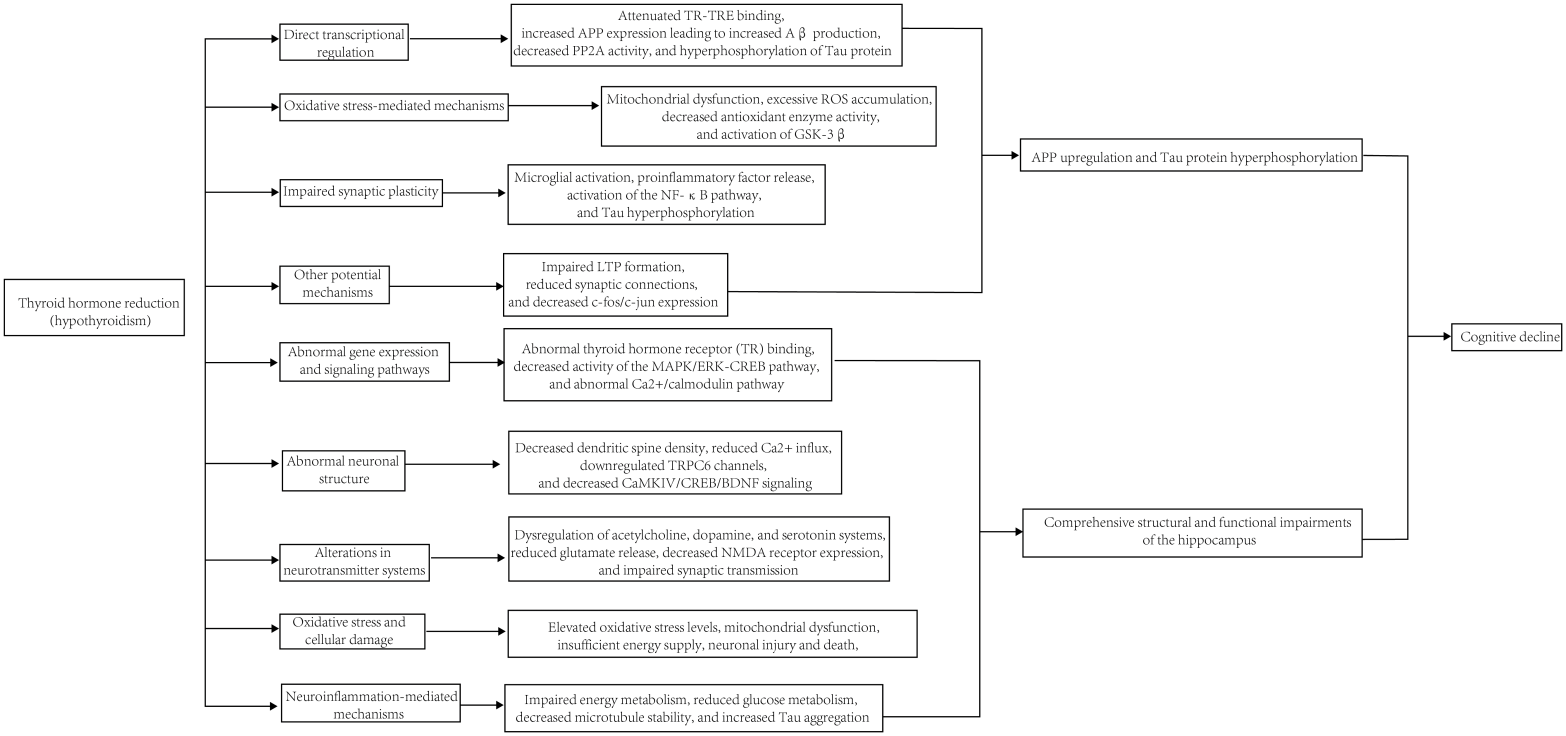
Mechanisms underlying thyroid hormone reduction-induced cognitive decline.

Hypothyroidism is a disease characterized by insufficient synthesis and secretion of thyroid hormones (THs) or reduced biological effects. It is defined as a condition in which thyroid stimulating hormone (TSH) levels are higher than the normal range and free T4 (FT4) levels are below the normal range (16). Primary hypothyroidism is a common disorder worldwide, with iodine deficiency and Hashimoto thyroiditis as the principal causes (17). Iodine deficiency is the leading cause of endemic goiter across the globe. Insufficient iodine intake impairs the synthesis and secretion of thyroid hormones, which not only triggers thyroid follicular hyperplasia and goiter, but also severely disrupts central nervous system function throughout all life stages. Populations living in iodine-deficient regions are at high risk of cognitive deficits. In particular, iodine deficiency during the embryonic and early childhood periods leads to irreversible damage to brain development, resulting in lifelong intellectual disability, memory decline and poor learning ability. For adults with long-standing iodine deficiency and goiter, mild impairments in attention, psychomotor speed and executive function are also commonly observed (3, 17). In the Whickham survey conducted in England, the prevalence of hypothyroidism in the general adult population was 7.5% in women and 2.8% in men (18). Overt hypothyroidism in adults can cause cognitive dysfunction, such as memory loss, decreased attention, and impaired executive function (19). A prospective, open-label interventional study demonstrated that patients with hypothyroidism exhibit significant deficits in spatial, associative, and verbal memory (20), with verbal memory being the most prominently affected cognitive domain (21). Therefore, understanding the relationship between hypothyroidism and cognitive function is of great significance for improving patients’ quality of life and well-being. Psychological and cognitive changes have been documented for many years in adults with altered thyroid function in both basic and clinical research. Thyroid hormones play important roles in the regulation of brain metabolism, myelination, cellular repair, and neurogenesis. The hippocampus is a key structure for learning and memory; it has a high density of thyroid hormone receptors and is highly sensitive to changes in thyroid hormone levels (22). A decrease in thyroid hormone levels alters the function and related structures of the hippocampus, focusing on the number of hippocampal cells in the brain structure (23), as well as a reduction in cerebral blood flow—particularly in regions mediating visual-spatial processing, attention, motor speed, and working memory (24). In addition, changes in the expression of certain enzymes and various genes can affect the activity of the central nervous system (CNS). The expression of amyloid precursor protein genes is associated with Alzheimer’s disease (AD) and cognitive function (25). In mice with hypothyroidism, a decrease in the synthesis and activity of acetyltransferase cholinergic neurons in the hippocampus has also been observed (14). What’s more, excessive phosphorylation of TAU, impaired expression of signal molecules related to synaptic plasticity and memory, and elevated levels of proinflammatory cytokines have been observed, which also highlights that hypothyroidism is an important risk factor for AD (26). As shown in **Figure 1**. In hypothyroidism, hippocampal Na+, K+-ATPase activity, and glutamate levels are also reduced, which could be involved in memory deficit in mice (27).

In clinical reports, studies have found significant impairment of attention, intelligence, memory, language, executive power, and sensory functions, as well as mental motor speed in patients with hypothyroidism (19). Smith et al. found that patients with severe hypothyroidism (median TSH: 83.2 mIU/L) experienced slower reaction speed, decreased fine motor ability of the hands, reduced performance on accelerated tests, and depressive symptoms (28). A prospective study, which compared the neuropsychological symptoms of patients with subclinical hypothyroidism, clinical hypothyroidism, and euthyroidism at baseline and after 3 to 6 months of levothyroxine (LT4) treatment, found that patients with overt hypothyroidism had spatial, associative, and verbal memory deficits closely related to the integrity of the hippocampus and frontal lobe regions (20). Compared with the euthyroid rats, thyroidectomized rats exhibited lower learning ability, as well as impaired long-term and short-term memory functions (29). Imaging studies using the Functional Magnetic Resonance Imaging of the Brain (FMRIB) integrated registration and segmentation tool (FIRST) have demonstrated significant volume reduction in the right hippocampus of hypothyroid patients (23). Functional magnetic resonance imaging (fMRI) revealed significant memory impairment—particularly in working memory—accompanied by changes in the bilateral medial prefrontal cortex, posterior cingulate cortex, and left inferior lobule (24). After supplementation with thyroid hormone, the decline in glucose metabolism was reversed (30). In adult hypothyroid rats, some hippocampus-related classic conditioning reflexes could be restored after LT4 replacement (29). Treating hypothyroidism may protect against cognitive dysfunction, particularly in memory, visuospatial organization, attention, and reaction time; however, it cannot be completely reversed. Symptoms of memory impairment, including spatial memory and associative memory, may improve but often persist with residual deficits (20, 24, 31). However, Van Vliet et al. evaluated the thyroid function of 74,565 elderly people aged 57–93 years and found no relationship between thyroid dysfunction and overall cognitive function, nor a consistent association between thyroid dysfunction and executive function, memory, or dementia risk (32). This result is mainly attributed to the insufficient sensitivity of the neuropsychological test battery, differences in cohort age distribution, and compensatory mechanisms in the elderly that may mask cognitive deficits. Meanwhile, there are also limitations such as missing FT4 data, single measurement of thyroid function, and methodological heterogeneity among studies. Therefore, inconsistencies remain regarding the relationship between hypothyroidism and cognitive function, and larger sample sizes and more sensitive measurement tools are needed to explore this relationship. For patients with hypothyroidism, their cognitive function should be closely monitored, and timely intervention and treatment should be provided. As a common sequela of endocrine surgery, thyroidectomy (partial or total) often induces iatrogenic hypothyroidism and subsequent cognitive dysfunction. Total thyroidectomy leads to irreversible thyroid hormone deficiency and more severe cognitive symptoms. Standard levothyroxine replacement can effectively alleviate most cognitive deficits by restoring TH levels and rescuing hippocampal synaptic function. Nevertheless, complete recovery is difficult to achieve in some patients due to defective T4-T3 conversion and long-term neural damage. Excessive TSH suppression after surgery will also bring additional cognitive risks (5, 6, 21, 26).

### 3.2 Subclinical hypothyroidism and cognition

Subclinical hypothyroidism (SCH) refers to a condition in which serum TSH levels are higher than the normal reference range, but FT4 levels remain within the normal range (33). TSH levels in the elderly tend to increase with age, leading to an increasing incidence of subclinical hypothyroidism (9). Data from the United States National Health and Nutrition Examination Survey (NHANES) showed a prevalence of 4.6%, with the vast majority of individuals (>90%) having subclinical rather than overt hypothyroidism (34). At present, cognitive dysfunction in patients with subclinical hypothyroidism is still observed in clinical practice and is associated with mild abnormalities in emotion and cognition (35). The inconsistent findings regarding subclinical hypothyroidism (SCH) and cognitive function may be largely attributed to variability in TSH cutoff values used across studies. Cognitive impairment is more evident in patients with TSH >10 mIU/L, whereas no consistent association is observed in those with TSH 4.5–10 mIU/L. Furthermore, the use of fixed adult TSH reference ranges fails to account for the physiological increase in TSH with advancing age, particularly in elderly populations, which further contributes to the controversy (32, 36). TSH levels are related to wedge-shaped connections in the default network, with slightly prolonged response time during working memory tasks and weakened memory and wedge-shaped integration ability (37). Spatial and verbal memory impairment was observed in SCH patients (P < 0.05) in a prospective study. After LT4 replacement therapy, the impaired spatial and verbal memory was improved to some extent (20). A meta-analysis including 12,315 individuals also demonstrated a close relationship between subclinical hypothyroidism and depression (38).

In addition, neuroelectrophysiological studies have shown longer P300 latency of auditory and visual evoked potentials and prolonged response time to visual stimuli in patients with subclinical hypothyroidism (39). A double-blind randomized experiment also indicated that the brain regions responsible for working memory were affected by subclinical hypothyroidism (40). But now, there is no sufficient evidence from randomized trials to confirm the impact of subclinical hypothyroidism treatment on dementia (9), and more studies are needed to further explore the relationship between subclinical hypothyroidism and cognitive function. However, recent data indicate that subclinical hypothyroidism (SCH) tends to specifically impair executive function, information processing speed and attention, while global memory function remains relatively intact. In contrast, generalized memory impairment is more commonly observed in overt hypothyroidism (41). Recent high-quality evidence from large randomized controlled trials and meta-analyses demonstrates that levothyroxine treatment does not significantly improve cognitive function or quality of life in patients with subclinical hypothyroidism (SCH), especially among older adults. These findings challenge the routine use of thyroid hormone replacement therapy in this population and highlight the ongoing clinical debate regarding the net benefits of such treatment. Although the TRUST trial confirmed that levothyroxine fails to alleviate symptoms or enhance quality of life in elderly patients with subclinical hypothyroidism, the study still has several limitations. Post-treatment free thyroxine levels were not routinely monitored; data on cardiovascular risk factors such as blood lipids were lacking; and the trial was underpowered to evaluate cardiovascular events and mortality. In addition, the accuracy of conventional immunoassays and the influence of levothyroxine administration timing on drug absorption remain controversial. These considerations indicate that further standardized, well-designed clinical trials are still needed to clarify the therapeutic value of levothyroxine in older adults with subclinical hypothyroidism (42, 43).

### 3.3 Overt hyperthyroidism and cognition

Hyperthyroidism is usually caused by excessive production of thyroid hormones, defined as TSH levels below the normal range, FT4 and FT3 above the normal (44). 73.3% of hyperthyroidism is caused by Graves (45). Overt thyrotoxicosis may cause heat intolerance, sweating, anxiety, and irritability, which can significantly affect normal daily life. As early as 1996, Stern et al. conducted a study of 37 patients with hyperthyroidism and found that they experienced anxiety, irritability, and impairments in memory, attention, planning, and working ability. Furthermore, although cognitive function improved somewhat after achieving euthyroidism, residual cognitive deficits remained. This has sparked interest in the relationship between hyperthyroidism and cognitive function (46). Some studies have reported the relationship between FT4 or TSH and cognitive dysfunction or dementia in both basic and clinical research. The mid-frontal, mid-occipital, and parieto-occipital regions are associated with the domains of working memory and executive function; a decrease in glutamine in white matter and occipital gray matter is associated with scores on cognitive tests of attention and concentration (47). Positron emission tomography (PET) scanning found irregular glucose metabolism in the limbic system of the right hemisphere, which is related to long-term memory (48). By altering the expression of certain genes (e.g., those involved in neurogenesis) and increasing neuronal death, hyperthyroidism can lead to a decrease in antioxidants and an increase in induced oxidative stress, thereby affecting cognitive function (10). Furthermore, Li et al. found significantly higher circulating total tau protein levels in patients with hyperthyroidism, which may be implicated in the pathogenesis of AD (15). In clinical researches, a meta-analysis including three case-control and eight cohort studies found that individuals with higher FT4 levels or those with TSH levels below the normal range have an increased risk of dementia (49). Using Wisconsin Card Sorting Test and N-back Test to evaluate working memory and executive function, Karolina et al. found that compared with healthy subjects, significant disturbances in working memory and executive function were observed in patients with hyperthyroidism, and these disturbances were associated with longer disease duration (50). A prospective longitudinal study focusing on 3,401 elderly men aged 70–89 years found, after follow up, that higher FT4 levels were closely related to dementia, and higher FT4 levels were one of the risk factors for dementia after adjusting for covariates (51). Large-scale registry-based date also found that the risk of dementia in patients with hyperthyroidism increased by 16% for every 6-month period of reduced TSH (52), further confirming the results of the above studies. A decrease in the peripheral conversion of T4 to T3, leading to a reduction in the T3/T4 ratio, indicates that T3 is closely related to the brain structure of patients with AD (53). In addition, animal experiments have also demonstrated the impact of hyperthyroidism. The hippocampus is a key region related to cognition, excitatory synaptic transmission is crucial in the process of learning and memory. In hyperthyroid mice, the number of mature dendritic spines in the hippocampal CAI region is significantly decreased, accompanied by reduced levels of AMPA- and NMDA-type glutamate receptors (12). These morphological and synaptic deficits in the hippocampal CA1 region are closely associated with behavioral performance in animal models. Impaired LTP and decreased dendritic spine density collectively result in obvious impairments in spatial learning and memory, as manifested by poor performance in the MWM(Morris Water Maze). In addition, CA1 neuronal atrophy and synaptic dysfunction also lead to declined recognition memory ability in the Novel Object Recognition Test, establishing a clear link between hippocampal structural damage, synaptic plasticity impairment and behavioral cognitive dysfunction (12). Hyperthyroidism induces excessive glutamate release in the hippocampus and subsequent excitotoxicity. The downregulation of NMDA/AMPA receptor subunits (GluN2A/B, GluA1/2) observed in relevant studies represents an early compensatory response to alleviate intracellular calcium overload, rather than irreversible pathological receptor loss (54). In the setting of prolonged hyperthyroidism, this adaptive regulation gradually progresses to pathological alterations, including irreversible receptor deficiency, dendritic spine atrophy and synaptic dysfunction, ultimately leading to cognitive impairment. There is a close bidirectional molecular crosstalk between thyroid hormones and the glutamatergic signaling system. Thyroid dysfunction, especially hyperthyroidism, disrupts this regulatory axis, manifested as enhanced glutamate synthesis and impaired glutamate clearance. This results in glutamate accumulation in the synaptic cleft and persistent overactivation of glutamate receptors. Conversely, overactivated glutamatergic excitatory signaling further disturbs central neuroendocrine homeostasis and reversely modulates the local actions of thyroid hormones in the brain (55). In a 12-week study involving 33 hyperthyroid patients, the cumulative effect of long-term elevated thyroid hormone levels can also increase the risk of dementia (41). In addition, prospective studies have found that memory, executive, visuospatial, and motor functions improve after treatment, and connectivity to the right frontoparietal network also increases—this is significantly positively correlated with object recognition memory scores (56). Long-term exposure to high thyroid hormone levels increases the risk of cardiovascular and cerebrovascular diseases, such as systolic hypertension and atrial fibrillation, which in turn elevates the risk of cognitive impairment and dementia (57). Thus, it is necessary to re-evaluate the impact of hyperthyroidism on cognitive function.

### 3.4 Subclinical hyperthyroidism and cognition

Subclinical hyperthyroidism is defined as a condition in which TSH levels are low but serum thyroid hormone levels are within the normal range, and patients may or may not be accompanied by mild hyperthyroid symptoms (58). With the improvement of detection technology and the application of TSH suppression therapy after thyroid cancer treatment, the incidence of subclinical hyperthyroidism is increasing. Subclinical hyperthyroidism is classified into endogenous and iatrogenic subtypes, which differ in their impacts on cognitive function. The endogenous subtype is mainly caused by Graves’ disease and other thyroid disorders, featured by fluctuating hormone levels, with severe cases (TSH < 0.1 mIU/L) accounting for 42%. The iatrogenic subtype is mostly induced by L-T4 suppressive therapy, presenting a stable condition in a dose-dependent manner, with severe cases accounting for 31% (59). The duration and stability of T4 levels are crucial to the severity of cognitive impairment. Long-term steadily elevated T4 and suppressed TSH in the iatrogenic subtype tend to lead to insidious and persistent cognitive damage, whereas the endogenous subtype is characterized by volatile hormone levels and inconsistent cognitive outcomes. Each additional six months of sustained TSH reduction increases dementia risk by 16%, with the highest risk observed in those with long-term exposure (≥ 5 years) (60). Long-term L-T4 suppression impairs cognitive function in a dose-dependent manner. Animal experiments have confirmed that sustained high T4 (mimicking the iatrogenic subtype) is more likely to damage the hippocampus and glutamate receptors than fluctuating hyperthyroidism (mimicking the endogenous subtype). Meta-analyses have shown that subclinical hyperthyroidism raises the overall risk of dementia by 38%, and recommend stratified research based on etiology and exposure duration, further highlighting the importance of distinguishing the two subtypes (61). As early as 2000, a prospective study first found that reduced TSH levels were associated with a more than threefold higher risk of dementia and Alzheimer’s disease, and that subclinical hyperthyroidism in the elderly increases the risk of dementia and Alzheimer’s disease (61). Lower TSH is a risk factor for dementia in elderly people, especially between 1.55-1.60 mIU/L (62). Thirty-one patients who received TSH suppression therapy showed significant impairment in executive function, psychomotor speed, and attention (63). A prospective study found that patients may experience short-term memory impairment, attention disorder, word selection disorder, and depression after TSH suppression. Moreover, it will worsen in word selection (64). Eleven prospective cohorts followed 16,805 patients and found that primary subclinical hyperthyroidism might be associated with an elevated risk of dementia (36). A prospective cohort study including 2,558 adults conclude that TSH < 0.10 mIU/L was associated with a higher risk of dementia and a larger cognitive decline in subclinical hyperthyroidism (65). By comparing the thyroid function status and cognitive abilities of 1,171 individuals, Ceresini et al. found that elderly individuals with subclinical hyperthyroidism had lower MMSE scores (66). After a 5-year follow-up, the authors found that lower-normal TSH levels within the normal range were independently associated with MCI and dementia (67). Considering the confounding effect of atrial fibrillation (AF), subclinical hyperthyroidism impairs cognitive function through dual mechanisms involving direct neurotoxic damage and indirect cardiovascular pathways (68). Excessive thyroid hormones exert direct detrimental effects on the hippocampus and disrupt glutamatergic signaling as well as synaptic plasticity. Meanwhile, subclinical thyroid dysfunction is closely associated with an elevated risk of new-onset atrial fibrillation. Atrial fibrillation contributes to the progression of cognitive decline mainly through multiple underlying mechanisms, including cerebral microembolism, chronic cerebral hypoperfusion, and persistent systemic inflammatory activation (69). Existing evidence indicates that even after accounting for the confounding influence of atrial fibrillation, the association between subclinical hyperthyroidism and cognitive impairment remains statistically significant, suggesting that AF serves as an important confounding and mediating factor in this relationship. Collectively, cognitive impairment related to subclinical hyperthyroidism arises from the combined effects of direct neuronal injury induced by thyroid hormone excess and indirect neurological damage mediated by atrial fibrillation and other vascular complications (70). Therefore, exploring the relationship between subclinical hyperthyroidism and cognitive impairment has really attracted everyone’s attention.

## 4 Conclusion

As one of the most important endocrine glands, the thyroid secretes hormones that are essential for metabolism, as well as the development, maturation, and function of the central nervous system. In a large number of studies, the effect of obvious thyroid dysfunction on cognitive function has been confirmed, and reasonable treatment can improve symptoms to some extent. However, cognitive impairment is not a characteristic feature of subclinical thyroid dysfunction. Although the prognosis of thyroid diseases is generally favorable, cognitive function remains critical for daily work and quality of life. Therefore, it is particularly important to recognize and explore the relationship between thyroid function and cognitive function. In clinical practice, timely diagnosis and intervention for individuals with cognitive impairment are of great significance. Furthermore, sensitive neuropsychological scales, rigorous statistical methods, prospective and double-blind intervention experiments, as well as large-sample studies, should be carried out. Reducing the incidence of cognitive impairment to decrease the medical burden, alleviate social pressure, and improve patients’ quality of life and well-being is of great significance.
